# A sex-specific difference in smartphone addiction, physical activity level, and cognitive functions among university students

**DOI:** 10.3934/publichealth.2026021

**Published:** 2026-03-27

**Authors:** Turki Abualait, Mohammad Ahsan

**Affiliations:** Department of Physical Therapy, College of Applied Medical Sciences, Imam Abdulrahman Bin Faisal University, Dammam, Saudi Arabia

**Keywords:** smartphone addiction, cognitive functions, physical activity, university students, gender differences

## Abstract

This study aimed to investigate the sex-specific differences in smartphone addiction, physical activity levels, and cognitive functions among university students. A cross-sectional survey was conducted with 256 university students aged 18–25. Participants completed questionnaires using the Smartphone Addiction Scale Short Version to assess their level of smartphone addiction. The working memory and selective attention domains of cognitive function were evaluated, and the International Physical Activity Questionnaire was used to determine participants' self-reported physical activity levels. The results revealed significant sex differences, with male students exhibiting higher levels of smartphone addiction (male = 46.42 ± 9.37; female = 38.27 ± 7.63) and greater physical activity (male = 3752 ± 1876; female = 3447 ± 1748) than their female counterparts. Additionally, female students demonstrated superior performance on selective attention tasks, including reaction time (female = 463.00 ± 50.53; male = 457.34 ± 59.31) and accuracy (female = 92.26 ± 6.53; male = 89.60 ± 8.39) across varied conditions, whereas no significant sex differences were observed in working memory or overall reaction time. These findings suggest that sex-specific factors may influence differences between male and female participants in smartphone use, cognitive function, and physical activity.

## 1. Introduction

Smartphone addiction has become a growing concern, particularly among university students, who are often characterized by high levels of smartphone use due to academic and social demands. For instance, studies have reported incidence rates of 29.14% among medical students in China and 41.2% among Moroccan nursing students, while other studies report similar figures across various regions and disciplines [1]–[4]. Such addiction is inherently linked to the pressures of academic life, where excessive reliance on smartphones for social interaction and study aids can lead to procrastination and a decline in overall academic performance [5]–[7].

Engaging in structured physical activities like exercise or sports has been linked to psychological benefits that may help reduce addiction-related behaviors [8]. Engaging in regular physical activity may serve as a preventive measure against smartphone addiction by providing an alternative form of engagement that promotes well-being and reduces screen time. Evidence suggests that physically active individuals may be less susceptible to smartphone addiction and enjoy better psychological resilience [9]. For instance, a systematic review found a significant correlation between higher levels of physical activity and fewer instances of smartphone addiction among university students, underscoring the protective effect that exercise may have against sedentary behaviors and related addictions [10]. A systematic review of randomized controlled trials found that both exercise and psychological interventions can effectively reduce smartphone addiction levels among university students. Findings from a cross-sectional study involving medical students illustrated significant associations between excessive smartphone use and various psychosocial factors. These associations emphasize the complex interplay between excessive smartphone use, physical inactivity, and mental health issues [6]. Therefore, it is essential to consider not only the role of physical activity but also the broader context of mental health to address smartphone addiction effectively. More engaging physical activity tends to reduce users' screen time and fosters healthier lifestyle patterns. However, existing literature nuances this relationship by indicating that other factors—such as resilience, social support, and the type and duration of smartphone use—may mediate the effects of physical activity on smartphone addiction [10],[11].

Cognitive functions, such as attention, memory, and executive function, are essential for academic success and overall well-being. Emerging evidence indicates that excessive smartphone use may negatively impact these cognitive domains, potentially hindering students' academic performance and personal development [12],[13]. Studies indicate that students who rely heavily on their smartphones often experience difficulties with attention, memory, and decision-making. A meta-analysis of 63 studies found a statistically significant yet modest negative association between technology use (including smartphone use) and students' academic performance. This suggests that increased smartphone and social media use may adversely affect the cognitive skills essential for academic success [6],[10]. Specific cognitive tasks have highlighted deficits in cognitive control among smartphone addicts. A study examining the Stroop effect—an indicator of cognitive control—found that students with higher smartphone addiction exhibited slower reaction times and made more errors compared to their peers with lower levels of addiction. This indicates that smartphone addiction may impair essential cognitive abilities, leading to reduced academic efficiency [12].

Smartphone addiction, often viewed as a behavioral addiction, has been shown to impact various demographic groups differently, particularly regarding sex. Research indicates that men tend to exhibit higher levels of smartphone addiction than women [14]. while other studies suggest that females have higher smartphone addiction than boys [15]. For instance, men report using smartphones more for entertainment and gaming, while women typically use smartphones primarily for communication and social networking [9],[16],[17]. Interestingly, some studies have even suggested that there is no difference in smartphone addiction between sexes [18]. These conflicting findings may stem from various factors. For instance, Su et al. noted that sex differences in smartphone addiction may be attributed to differences in economic and internet availability across countries [19]. Moreover, the implications of smartphone addiction extend to physical activity levels. Studies indicate that excessive smartphone use often correlates with a more sedentary lifestyle. In particular, young adults of both sexes show a clear trend: increased screen time is inversely associated with time spent on physical activity [16],[17]. Notably, women may experience this impact differently due to the added challenge of balancing societal expectations regarding physical appearance and health. While both sexes may struggle with the repercussions of a sedentary lifestyle, women generally face additional pressures related to body image, which can influence their motivation to engage in physical activity [9]. Cognitive functions, another critical area affected by both smartphone use and physical activity, have been shown to differ by sex. Research suggests that excessive smartphone use can reduce cognitive functions, including attention span and memory. Men often report greater cognitive decline associated with smartphone use, possibly due to engagement patterns that emphasize gaming and high-stimulation tasks [16].

To date, limited research has comprehensively examined the sex-specific differences between smartphone addiction, physical activity, and cognitive functions among university students. Understanding these sex-specific dynamics is crucial to developing targeted interventions that promote healthy technology use and lifestyle behaviors within this population. This study aimed to investigate the sex-specific differences in smartphone addiction, physical activity, and cognitive functions among university students. The findings of this study can contribute to the development of evidence-based strategies to address smartphone-related challenges and promote overall well-being among university students.

## 2. Materials and methods

### 2.1. Study design and setting

To achieve its objectives, this study adopted a cross-sectional comparative study design.

### 2.2. Ethical approval

The Deanship of Scientific Research at Imam Abdulrahman Bin Faisal University approved the study (IRB–2025–03–0100), and all participants provided informed consent.

### 2.3. Sample size

We performed a power analysis using G*Power software v3.1.9.4 (Franz Faul, Universität Kiel, Germany) and determined that a sample size of 254 participants (127 males and 127 females) would provide 90% statistical power at a 1% significance level (two-sided test) for a medium effect size (d = 0.50) with allocation ratio N2/N1 = 1.

### 2.4. Participants

A study was conducted among 254 university students (127 males and 127 females), aged 18–25. Participants were recruited from various academic programs at the university level. Students with any psychiatric or neurological disorders or disability were excluded from the study.

### 2.5. Measures

#### 2.5.1. Smartphone addiction

The level of smartphone addiction among participants was determined using the Smartphone Addiction Scale (SAS-SV) short version [20]. The SAS-SV, a 10-item self-report measure, assesses various aspects of smartphone use, including cyberspace-oriented relationships, daily-life disturbance, withdrawal, overuse, and tolerance. A six-point Likert scale, with 1 denoting “strongly disagree” and 6 denoting “strongly agree”, is used to evaluate it. The scale's lowest possible score is 10, and its highest potential score is 60. Users are categorized as either non-addicted if their scores are less than 31 for males and less than 33 for females, or as addicted if their scores are equal to or higher than 31 for males and equal to or higher than 33 for females. The Cronbach's alpha for the SAS-SV in this study was 0.78, indicating good internal consistency.

#### 2.5.2. Cognitive functions

The Psychology Experiment Building Language (PEBL) test battery was used to evaluate the cognitive function domains of working memory and selective attention. A flexible research tool for examining individual variation in neurocognitive function, the PEBL is an open-source software system for planning and conducting psychological studies. It has shown strong validity and dependability [21]. The battery is available for free download from the following website: http://pebl.sourceforge.net. However, the working memory and selective attention domains were evaluated in the PEBL battery using the memory automaticity and Flanker tasks, respectively.

#### 2.5.3. Physical activity

Participants' self-reported levels of physical activity were determined using the International Physical Activity Questionnaire (IPAQ). In addition to measuring overall physical activity in metabolic equivalents (METs) per minute and per week, this tool quantifies the intensity of various forms of physical activity and the time spent sitting. In addition to a summed score representing total activity level, the items are designed to yield distinct scores for walking, moderate-intensity activity, and high-intensity activity. It has been created and tested for use with adults aged 15–69 [22].

### 2.6. Statistical analysis

Descriptive statistics were calculated for all study variables. Results are presented as mean and standard deviation (SD), mean rank, standardized test statistic, and asymptotic significance (2-sided test). The cognitive functions of males and females, and addicted and non-addicted, were compared using a Mann–Whitney U test with non-normally distributed data (Shapiro–Wilk test, p > 0.05). An independent t-test was used to compare the anthropometric characteristics and outcome measures between males and females. A chi-squared test was applied to determine the differences between addicted and non-addicted participants for physical activity levels. A probability lower than 0.05 (p < 0.05) indicated statistical significance. All data analyses were performed using the IBM Statistical Package for the Social Sciences (IBM SPSS), version 26.0, for Windows (Armonk, New York, United States).

## 3. Results

**Table 1.** Descriptive statistics of anthropometric characteristics and outcome measures by sex.

	Sex	N	Mean	Std. Deviation	t	p	d
Age	Male	127	20.22	2.83	0.48	0.630	0.06
	Female	127	20.05	2.79			
Height	Male	127	168.35	7.24	2.23	0.026	0.03
	Female	127	166.53	5.70			
Weight	Male	127	69.30	8.97	3.46	<0.001	0.43
	Female	127	66.15	4.94			
Body mass index	Male	127	24.37	1.92	2.29	0.022	0.29
	Female	127	23.86	1.60			
Smartphone addiction	Male	127	46.42	9.37	7.60	<0.001	0.95
	Female	127	38.27	7.63			
Addicted	Male	83	62.52	14.40	−10.58	<0.001	1.73
	Female	72	42.12	8.32			
Non-Addicted	Male	44	44.12	11.90	−6.96	<0.001	1.34
	Female	55	32.42	3.36			
Physical activity (MET/min)	Male	127	3752	1876	1.34	0.18	0.16
	Female	127	3447	1748			

Table 1 presents descriptive statistics for anthropometric characteristics, smartphone addiction, and physical activity levels for male and female participants. Smartphone addiction prevalence was 61% (165/254), with males (65.35%) showing a higher rate than females (56.69%). There are significant differences between male and female participants in height, weight, body mass index, and smartphone addiction. There were no significant differences between males and females in age or physical activity.

**Table 2.** Comparison between smartphone addiction and physical activity levels among all participants.

Smartphone addiction	Physical activity levels			
	Low	Moderate	High	N	*χ* ^2^	p
Addicted	82	51	0	133	120.181	<0.001
Non-Addicted	10	48	63	121		
Total	92	99	63	254		

Table 2 presents inferential statistics for the association between smartphone addiction and physical activity levels among all participants. Table 2 shows a significant difference (*χ*^2^ = 120.18, p < 0.001) between smartphone addicted and non-addicted participants for various physical activity levels.

**Table 3.** Inferential statistical analysis for cognitive function between males and females.

	Sex	Mean rank	Mann–Whitney U	Asymp. Sig. (2-tailed)
Reaction time	Male	125.83	7843.00	0.558
	Female	131.25		
Accuracy	Male	115.68	6523.50	0.005*
	Female	141.73		
Response time varied level 1–2	Male	130.52	7927.00	0.657
	Female	126.41		
Accuracy varied level 1–2	Male	108.80	5629.50	0.000*
	Female	148.82		
Response time varied level 3–4	Male	115.74	6531.00	0.005*
	Female	141.67		
Accuracy varied level 3–4	Male	110.31	5825.00	0.000*
	Female	147.27		
Response time consistent level 1–2	Male	123.77	7575.00	0.299
	Female	133.38		
Accuracy consistent level 1–2	Male	120.03	7088.50	0.057
	Female	137.24		
Response time consistent level 3–4	Male	121.82	7321.00	0.142
	Female	135.40		
Accuracy consistent level 3–4	Male	106.90	5382.50	0.000*
	Female	150.78		

Note: *p < 0.05.

Table 3 indicates that there were no significant differences in reaction time between male and female participants (MWU = 7843.00, p = 0.558), whereas accuracy differed significantly between the two groups (MWU = 6523.50, p = 0.005): Accuracy Varied Level 1–2 (MWU = 5629.50, p = 0.000), Response Time Varied Level 3–4 (MWU = 6531.50, p = 0.005), Accuracy Varied Level 3–4 (MWU = 5825.00, p = 0.000), and Accuracy Consistent Level 3–4 (MWU = 5382, p = 0.000).

Table 4 shows significant differences between addicted and non-addicted participants for accuracy (MWU = 6325.50, p = 0.002), Accuracy at the Varied Level 3–4 (MWU = 6965.50, p = 0.040), and Response Time Consistent with Level 3–4 (MWU = 6871.00, p = 0.027), whereas no significant differences existed between addicted and non-addicted participants for other cognitive function parameters.

Table 5 indicates that there are no significant differences among participants with low, moderate, and high physical activity levels in cognitive function parameters, except for accuracy, which shows a significant difference.

**Table 4.** Inferential statistical analysis of cognitive functions among smartphone addicted and non-addicted participants.

	Smartphone	Mean rank	Mann–Whitney U	Asymp. Sig. (2-tailed)
Reaction time	Addict	131.47	7785.00	0.505
	Non-addict	125.29		
Accuracy	Addict	142.44	6325.50	0.002*
	Non-addict	113.43		
Response time varied level 1–2	Addict	124.62	7664.00	0.384
	Non-addict	132.69		
Accuracy varied level 1–2	Addict	136.11	7167.50	0.085
	Non-addict	120.27		
Response time varied level 3–4	Addict	123.95	7574.00	0.306
	Non-addict	133.42		
Accuracy varied level 3–4	Addict	137.63	6965.50	0.040*
	Non-addict	118.63		
Response time consistent level 1–2	Addict	125.27	7750.00	0.468
	Non-addict	131.99		
Accuracy consistent level 1–2	Addict	128.90	8126.50	0.927
	Non-addict	128.07		
Response time consistent level 3–4	Addict	118.66	6871.00	0.027*
	Non-addict	139.14		
Accuracy consistent level 3–4	Addict	132.44	7656.00	0.373
	Non-addict	124.24		

Note: *p < 0.05.

**Table 5.** Inferential statistical analysis of cognitive functions among participants with low, moderate, and high physical activity levels.

	PA	N	Mean rank	Chi-square	Asymp. Sig.
Reaction time	Low	92	123.80	2.407	0.300
	Moderate	98	137.39		
	High	64	121.36		
Accuracy	Low	92	147.57	10.501	0.005
	Moderate	98	122.22		
	High	64	110.91		
Response time varied level 1–2	Low	92	129.38	0.808	0.668
	Moderate	98	123.90		
	High	64	134.42		
Accuracy varied level 1–2	Low	92	135.70	1.912	0.385
	Moderate	98	127.80		
	High	64	119.25		
Response time varied level 3–4	Low	92	128.65	0.034	0.983
	Moderate	98	129.26		
	High	64	127.09		
Accuracy varied level 3–4	Low	92	131.18	3.478	0.176
	Moderate	98	135.37		
	High	64	113.91		
Response time consistent level 1–2	Low	92	136.52	2.016	0.365
	Moderate	98	121.34		
	High	64	128.16		
Accuracy consistent level 1–2	Low	92	123.65	3.384	0.184
	Moderate	98	138.71		
	High	64	119.52		
Response time consistent level 3–4	Low	92	127.75	0.028	0.986
	Moderate	98	129.46		
	High	64	128.08		
Accuracy consistent level 3–4	Low	92	136.60	5.056	0.080
	Moderate	98	132.32		
	High	64	110.89		

## 4. Discussion

This study aims to investigate the sex-specific effects of smartphone addiction and physical activity levels on cognitive functions among university students. The results showed no significant difference in reaction time between male and female participants, whereas accuracy differed significantly between the two groups. Additionally, the findings indicate significant differences in accuracy among addicted and non-addicted participants at the varied levels of 3–4, along with response times consistent with those levels. However, no significant differences were found for other cognitive function parameters between addicted and non-addicted participants. Furthermore, the results reveal no significant differences in cognitive function parameters across participants with low, moderate, and high physical activity levels, except for accuracy, which differs significantly. This study provides insights into the sex-specific differences in smartphone addiction, cognitive functions, and physical activity among university students. The findings suggest that male students exhibit higher levels of smartphone addiction and physical activity compared to their female counterparts, while female students showed higher performance in selective attention tasks. Notably, both male (46.4 ± 9.37) and female (38.3 ± 7.63) participants scored above the established clinical cutoffs for smartphone addiction (31 for males, 33 for females), indicating a high prevalence of problematic smartphone use across the entire sample. This finding highlights a broader public health concern that extends beyond sex-specific differences and underscores the need for university-wide interventions targeting excessive smartphone use. Although some cognitive parameters reached statistical significance, effect sizes were small (e.g., d < 0.20 for general reaction time), suggesting that these differences may not be clinically meaningful. Future research should consider both statistical and practical significance when interpreting cognitive performance across sexes.

The influence of sex on smartphone addiction is increasingly well-documented in the literature, with varying prevalence rates highlighted across different demographics and contexts. One meta-analysis, including 19 studies, reported a smartphone addiction prevalence of 41.93% among Asian medical students, revealing that female medical students are often at a higher risk of smartphone addiction, as their rates were reported at 39.75%, contrasting with 36.25% for male counterparts. This suggests that female students may exhibit higher emotional attachment to smartphone use, which aligns with findings that women are more likely to use smartphones for social connections, potentially reflecting a greater vulnerability to addiction [23]. Taywade and Khubalka observed that male participants primarily use smartphones for entertainment, whereas female participants primarily use them for communication [24]. Females tend to be more active than males in online interpersonal interactions and information exchange [25]. In contrast, males are more inclined to engage in online activities like gaming, shopping, and sexual searches. Nonetheless, increased internet access among females is expected to narrow the gender gap in internet use in the future [26]. The systematic meta-analysis conducted by Wan et al. demonstrated a significant moderating effect of sex on mobile phone addiction, where males showed greater reliance on smartphones but also reported higher levels of social support that mitigated addiction tendencies [27]. This underscores the paradox that while males may exhibit higher addiction levels, supportive environments can help manage addiction rates. Conversely, Alimoradi et al. showcased that both males and females experienced elevated smartphone addiction levels during lockdown periods, especially as social media usage surged [28]. This increase, however, did not differentiate significantly by sex in this context, raising questions about when and how sex differences manifest in addiction patterns. The authors noted peer-reviewed studies documenting the prevalence of 30.7% for smartphone addiction, with males showing a consistent temporal increase throughout the pandemic. Zheng et al. addressed the intersection of sex nonconformity and problematic smartphone use among Chinese adolescents, finding a significant positive association, particularly amongst male adolescents exhibiting sex nonconformity [29]. This underscores that those deviating from traditional sex norms may disproportionately engage in behaviors leading to smartphone addiction, suggesting a unique vulnerability attributed to social pressures that can vary by sex. Multiple systematic reviews have suggested a notable prevalence of smartphone addiction among specific demographics, particularly among medical students in Asia, where prevalence rates can range as high as 41.93% [23]. This data reflects a larger trend wherein male students were found to have higher addictive scores on mobile use, which aligns with the findings in other studies emphasizing males' greater vulnerability to gaming and internet addiction [30],[31].

In the systematic review by of the total 5497 medical students analyzed, a striking 67.5% of those affected by smartphone addiction were female, which indicates significant sex differences in susceptibility [23]. Furthermore, cognitive deficits associated with problematic internet use (PIU) are distinctly pronounced across sexes. A meta-analysis conducted in 2019 and involving 2922 participants found similar neurocognitive impairments, such as deficits in inhibitory control, working memory, and decision-making, which are critically affected by excessive internet use, with these effects being modulated by the type of PIU [32]. Sex differences in the context of smartphone addiction and physical activity reveal striking trends discerned through empirical research. A systematic review evaluating the effects of exercise and psychological interventions on smartphone addiction [9]. Among the 23 studies analyzed, it was noted that interventions tailored to university students, particularly females who displayed greater susceptibility to smartphone addiction, were more impactful. Approximately 60% of students reported significant improvements following interventions, underscoring the crucial need to prioritize sex-specific approaches in future research and practice.

Nikolic et al. offered compelling insights into the interrelationships between smartphone addiction, physical activity levels, and mental health among medical students [6]. The study surveyed 761 students and reported a 21.7% prevalence of smartphone addiction, with slightly higher rates among males (22.9%) compared to females (21.1%). Although male students exhibited higher addiction scores, multivariate regression analysis revealed that female students displayed more pronounced psychological detriments associated with their smartphone use, signifying a critical area for intervention: females demonstrated a higher likelihood of correlational factors such as elevated stress, anxiety, and depression linked to smartphone addiction. The cumulative evidence posits a clear narrative: not only is smartphone addiction prevalent among students, but it also exhibits significant sex disparities, with females being more adversely affected in terms of psychological outcomes [33]. A study revealed that gender differences in smartphone addiction could be attributed to the differences in economic and internet availability in different countries. This is because stronger economies are often associated with higher internet penetration rates, and greater internet availability may increase the likelihood of smartphone addiction [19]. Additionally, economic development is closely linked to gender equality, which encompasses digital inclusion. As the GDP per capita and internet penetration rate of a country increase, gender differences in internet-related addictive behaviors, including smartphone addiction, also tend to decrease. In particular, the detrimental implications of smartphone addiction extend to engagement in physical activities, presenting a multifaceted public health challenge. These studies' limitations primarily involve reliance on self-reported measures, which can introduce bias and limit the replicability of findings. Furthermore, cross-sectional designs predominate in the literature, revealing associations but undermining the ability to infer causation. Consequently, future longitudinal studies employing rigorous methodologies could more effectively illuminate causal pathways.

This study adopted a cross-sectional study design, which limits its ability to establish causal relationships. The term smartphone addiction has remained limited to prior definitions or synonyms. This study did not assess the measurement invariance of the Smartphone Addiction Scale Short Version (SAS-SV) across sexes. Therefore, observed differences in smartphone addiction scores between males and females may reflect measurement artifacts rather than true behavioral disparities. Future studies should establish the psychometric equivalence of the SAS-SV across demographic groups before drawing comparative conclusions. Longitudinal research is needed to examine the long-term effects of smartphone addiction on cognitive functions and physical activity, as well as potential sex-specific trajectories. The sex differences observed in this study may be attributed to sociocultural factors, including differences in social expectations, emotional regulation, and coping strategies. These findings underscore the need for targeted interventions that address sex-specific challenges and promote healthy technology use and balanced lifestyles among university students. Furthermore, future studies could investigate the role of additional factors, such as academic stress, social support, and personality traits, in shaping the sex-specific patterns observed in this study.

## 5. Conclusions

This study, in line with others, provides evidence of sex-specific differences in smartphone addiction, cognitive functions, and physical activity among university students. Male students exhibited higher levels of smartphone addiction and physical activity, while female students demonstrated higher performance in selective attention tasks. These findings underscore the need for targeted interventions that address the distinct challenges faced by male and female university students regarding smartphone use, cognitive development, and physical well-being.

## Use of AI tools declaration

To complete this study, Mendeley was used for referencing, and Grammarly for paraphrasing and grammatical corrections.
